# Resultados de médio e longo prazo do tratamento endovenoso de varizes com *laser* de diodo em 1940 nm: análise crítica e considerações técnicas

**DOI:** 10.1590/1677-5449.010116

**Published:** 2017

**Authors:** Luiz Marcelo Aiello Viarengo, Gabriel Viarengo, Aline Meira Martins, Marília Wechellian Mancini, Luciana Almeida Lopes

**Affiliations:** 1 Clínica Viarengo – CV, Jundiaí, SP, Brasil.; 2 Núcleo de Pesquisa e Ensino de Fototerapia nas Ciências da Saúde – NUPEN, São Carlos, SP, Brasil.

**Keywords:** laser, varizes, terapia a *laser*, técnicas de ablação

## Abstract

**Contexto:**

Desde a introdução do *laser* endovenoso para tratamento das varizes, há uma busca pelo comprimento de onda ideal, capaz de produzir o maior dano seletivo possível com maior segurança e menor incidência de efeitos adversos.

**Objetivos:**

Avaliar os resultados de médio e longo prazo do *laser* de diodo de 1940 nm no tratamento de varizes, correlacionando os parâmetros utilizados com a durabilidade do desfecho anatômico.

**Métodos:**

Revisão retrospectiva de pacientes diagnosticados com insuficiência venosa crônica em estágio clínico baseado em clínica, etiologia, anatomia e patofisiologia (CEAP) C2 a C6, submetidos ao tratamento termoablativo endovenoso de varizes tronculares, com *laser* com comprimento de onda em 1940 nm com fibra óptica de emissão radial, no período de abril de 2012 a julho de 2015. Uma revisão sistemática dos registros médicos eletrônicos foi realizada para obter dados demográficos e dados clínicos, incluindo dados de ultrassom dúplex, durante o período de seguimento pós-operatório.

**Resultados:**

A média de idade dos pacientes foi de 53,3 anos; 37 eram mulheres (90,2%). O tempo médio de seguimento foi de 803 dias. O calibre médio das veias tratadas foi de 7,8 mm. A taxa de sucesso imediato foi de 100%, com densidade de energia endovenosa linear (*linear endovenous energy density*, LEED) média de 45,3 J/cm. A taxa de sucesso tardio foi de 95,1%, com duas recanalizações por volta de 12 meses pós-ablação. Não houve nenhuma recanalização nas veias tratadas com LEED superior a 30 J/cm.

**Conclusões:**

O *laser* 1940 nm mostrou-se seguro e efetivo, em médio e longo prazo, para os parâmetros propostos, em segmentos venosos com até 10 mm de diâmetro.

## INTRODUÇÃO

A fototermoablação endovenosa (*endovenous laser ablation*, EVLA) emergiu como padrão no tratamento da insuficiência venosa em vários centros espalhados pelo mundo e como uma alternativa minimamente invasiva no tratamento de varizes tronculares. Desde então, houve uma busca contínua pelo comprimento de onda ideal capaz de produzir o maior dano seletivo possível com maior segurança e menor incidência de eventos adversos. Diferentes comprimentos de ondas e diferentes tipos de fibras ópticas foram testados com essa finalidade^1^^-^ 8. Vários autores demonstraram que todos os comprimentos de ondas utilizados para tratar varizes foram igualmente capazes de produzir os resultados anatômicos desejados. A diferença fundamental no resultado entre os diferentes comprimentos de ondas foi relacionada à ocorrência de eventos adversos^9^^-^ 11.

Do ponto de vista da física, quanto maior o coeficiente de absorção da luz por um tecido ou cromóforo, maior é a quantidade de calor gerado e maior o confinamento da zona de geração de calor. Esses princípios apontariam uma vantagem do *laser* de 1940 nm, com atuação no segundo pico de maior absorção pela água, tornando o procedimento teoricamente mais efetivo e mais seguro.

A durabilidade é uma importante característica de longo prazo de todos os procedimentos vasculares. No que diz respeito ao tratamento de varizes, a recorrência é potencialmente uma medida valiosa do resultado de médio e longo prazo das diferentes modalidades de tratamento da doença venosa^12^^-^ 14.

Esse estudo tem por finalidade analisar, retrospectivamente, os resultados de médio e longo prazo do tratamento endovenoso de varizes com *laser* de diodo de 1940 nm, correlacionando os parâmetros utilizados no tratamento com a durabilidade do desfecho anatômico (oclusão fibrótica ou recanalização), melhora clínica na classificação CEAP e eventos adversos.

## MÉTODOS

Foi realizada uma revisão retrospectiva de pacientes diagnosticados com insuficiência venosa crônica em estágio clínico C2 a C6 na classificação baseada em clínica, etiologia, anatomia e patofisiologia (CEAP), submetidos ao tratamento endovenoso de varizes tronculares, em um único centro, com *laser* com comprimento de onda em 1940 nm (Medilaser, DMC, São Carlos, SP, Brasil), registro na Agência Nacional de Vigilância Sanitária nº 80030810129), com fibra óptica de emissão radial, no período de abril de 2012 a julho de 2015. O objetivo foi analisar os resultados de médio e longo prazo com relação ao desfecho anatômico (oclusão fibrótica, recanalização parcial e recanalização completa), melhora clínica na classificação CEAP e eventos adversos (pigmentação, tromboflebite, trombose venosa, parestesias, cordão fibrótico e outros). Todos os dados foram de-identificados.

Os sujeitos da população do estudo foram inicialmente identificados através de busca nos arquivos eletrônicos de pacientes com diagnóstico de varizes, insuficiência venosa ou hipertensão venosa, com ou sem ulceração, que tenham sido submetidos a ablação endovenosa de varizes tronculares com *laser* pelo menos 12 meses antes e que tenham realizado os controles clínicos e ultrassonográficos conforme a rotina estabelecida pelo serviço (7 dias, 30 dias, 3 meses, 6 meses, 9 meses, 12 meses, 18 meses, 24 meses e anualmente). Pacientes com síndrome pós-trombótica prévia ao tratamento cirúrgico ou com refluxo no sistema venoso profundo foram excluídos.

Após a aprovação do Comitê de Ética em Pesquisa (parecer nº 1.693.514), uma revisão sistemática dos registros médicos eletrônicos foi realizada para obter dados demográficos e dados clínicos, incluindo dados de ultrassom dúplex, durante o período de seguimento pós-operatório.

Os dados demográficos incluíram idade, gênero, raça e comorbidades. Os dados clínicos incluíram data do procedimento, diagnóstico clínico (classificação CEAP) pré-operatório, segmento venoso tratado por fototermoablação, extensão e calibre médio do segmento venoso tratado, procedimentos associados, dor pós-operatória mensurada por escala visual analógica (EVA), intercorrências intraoperatórias, intercorrências pós-operatórias imediatas (até 30 dias), intercorrências tardias com data da constatação e evolução, parâmetros de *laser* empregados (potência, total de energia e densidade de energia endovenosa linear, LEED), classificação clínica CEAP na última revisão do seguimento clínico dentro do período de estudo, data da última revisão para cálculo do tempo de seguimento em dias e, adicionalmente, informações sobre os achados ultrassonográficos obtidos na última revisão realizada no período de estudo, relativos ao aspecto da veia ablacionada (fibrótica, fibroelástica, trombótica, completamente recanalizada, parcialmente recanalizada, extensão do segmento recanalizado, competência ou não das junções safeno-poplítea e safeno-femoral).

### Análise estatística

Os dados foram compilados em tabela Excel® 2011, versão 14.3.6 (Microsoft, Redmond, WA, EUA), e analisados com o programa Bioestat, versão 5.3 (Instituto Mamirauá, Belém, PA, Brasil). As variáveis categóricas foram apresentadas em tabelas de contingência contendo valores absolutos e relativos. As variáveis quantitativas foram analisadas por estatística descritiva e expressas como média, desvio padrão (DP) e valor máximo e mínimo. A análise das diferenças dentro do grupo foi realizada com o teste *t* de Student. Foi considerado estatisticamente significante um valor de p < 0,05.

## RESULTADOS

Entre abril de 2012 e julho de 2015, foram identificados 152 pacientes submetidos ao tratamento termoablativo de varizes tronculares com *laser* de diferentes comprimentos de ondas. Todas as intervenções foram realizadas por um único cirurgião com larga experiência em EVLA, em regime ambulatorial extra-hospitalar, com anestesia perivenosa tumescente ecoguiada associada ao bloqueio do nervo femural orientado por ultrassom. Desse total, 50 membros em 50 pacientes foram tratados com laser de 1940 nm. Entre os 50 casos elegíveis para o propósito desse estudo, nove foram excluídos, sendo dois por apresentarem *follow-up* registrado inferior a 12 meses, quatro por apresentarem síndrome pós-trombótica prévia ao tratamento e três por seguimento inadequado e dados incompletos.

Trinta e sete pacientes (90,2%) eram mulheres e quatro (9,8%) eram homens, com média de idade de 53,3 anos (mínimo: 30 anos; máximo: 74 anos; DP: 12,4 anos). Entre as mulheres, o número médio de gestações foi de 2,2 (mínimo: 0; máximo: 9 gestações; DP: 1,71). Todos os dados demográficos estão sintetizados na Tabela 1.

**Tabela 1:** Dados demográficos dos pacientes (n = 41).

**Média de idade (variação), anos**	**53,3 (30-74)**
Gênero	
Feminino	37 (90,2%)
Masculino	4 (9,8%)
Raça	
Brancos	34 (82,9%)
Negros	7 (17,1%)
IMC (variação)	26,1 (18,6-43,1)
História familiar de doença venosa	36 (87,8%)
Tabagismo	4 (9,8%)
Dislipidemia	7 (17,1%)
Diabetes	3 (7,3%)
HAS	7 (17,1%)
Obesidade	5 (12,2%)

IMC: índice de massa corporal; HAS: hipertensão arterial sistêmica. Os dados são apresentados como números (%), salvo indicação em contrário.

Foram tratadas 34 veias safenas magnas (VSMs) (82,9%) e sete veias safenas parvas (VSPs) (17,1%) em 41 membros de 41 pacientes, sendo 19 membros do lado direito (16 VSMs e 3 VSPs) e 22 membros do lado esquerdo (18 VSMs e 4 VSPs). A classificação clínica CEAP pré-operatória está sumarizada na Tabela 2. Os parâmetros anatômicos dos segmentos venosos tratados e os parâmetros de *laser* empregados estão sumarizados nas Tabelas 3 e 4, respectivamente.

**Tabela 2:** Classificação clínica pré-operatória (CEAP).

	**C1**	**C2**	**C3**	**C4**	**C5**	**C6**
Geral (n = 41)	0	19	7	10	0	5
VSM* (n = 34)	0	18	6	8	0	2
VSP** (n = 7)	0	1	1	2	0	3

CEAP: clínica, etiologia, anatomia e patofisiologia; VSM: veia safena magna; VSP: veia safena parva.

* Pacientes com insuficiência exclusiva da VSM;

** Pacientes com insuficiência exclusiva da VSP.

**Tabela 3:** Parâmetros anatômicos das veias ablacionadas.

	**Ø médio (mm)**	**Variação (mm)**	**DP**	**Extensão ablacionada (cm)**	**Variação (cm)**	**DP**
VSM	7,82	(6,2 a 10,4)	1,08	48,1	(29 a 80)	11,9
VSP	7,14	(5,0 a 10,5)	2,03	24,3	(15 a 34)	6,2

VSM: veia safena magna; VSP: veia safena parva; DP: desvio padrão.

**Tabela 4:** Parâmetros de tratamento a *laser*.

	**Potência média (W)**	**Variação (W)**	**DP**	**LEED média (J/cm)**	**Variação (J/cm)**	**DP**
VSM	4,1	(3,5 a 7,0)	0,6	45,5	(15 a 104,7)	15,9
VSP	3,86	(3,0 a 4,0)	0,38	45,9	(35,8 a 54,6)	7,52
Global	4,06	(3,0 a 7,0)	0,57	45,3	(15 a 104,7)	14,8

VSM: veia safena magna; VSP: veia safena parva; DP: desvio padrão; LEED: densidade de energia endovenosa linear.

A ablação primária foi obtida em 100% dos casos. Como procedimento associado, foi realizada flebectomia de tributárias e ramos varicosos em todos os pacientes. Não houve nenhuma intercorrência intraoperatória.

O tempo médio de seguimento pós-operatório foi de 803 dias, variando de 467 a 1.360 dias (DP: 291,3).

A dor intraoperatória, mensurada por EVA variando de 0 a 10 (onde 0 corresponde ao estado “sem dor” e 10 a “dor intensa”), foi “sem dor” em 23 pacientes (56,1%), “dor leve” (até 3 pontos na EVA) em 17 pacientes (41,5%) e “dor moderada” (4 a 6 pontos na EVA) em um paciente (2,4%). Em todos os casos, a dor esteve relacionada à flebectomia.

Durante o período de seguimento, foram observadas duas recanalizações, sendo uma recanalização em toda a extensão da VSM (caso 1), detectada aos 421 dias de pós-operatório; e uma recanalização no segmento infragenicular da VSM (caso 2), detectada aos 342 dias de pós-operatório (Tabela 5).

**Tabela 5:** Casos com recanalização pós-operatória tardia.

	**Tempo (dias)**	**CEAP**	**Veia tratada**	**Diâmetro** **médio (mm) PO**	**Potência utilizada (W)**	**LEED** **média** **(J/cm)**	**IMC**
Caso 1	421	C3	VSM	8,1	3,5	19,0	28,1
Caso 2	342	C6	VSM	9,0	5,0	15,0	29,6

VSM: veia safena magna; IMC: índice de massa corporal; PO: pré-operatório.

Não foi observada nenhuma recanalização ou falha de tratamento nas VSPs. Nas veias safenas tratadas com sucesso anatômico (VSM + VSP), a LEED média foi de 46,8 J/cm (variando de 30,7 J/cm a 104,7 J/cm).

**Figura 1:**
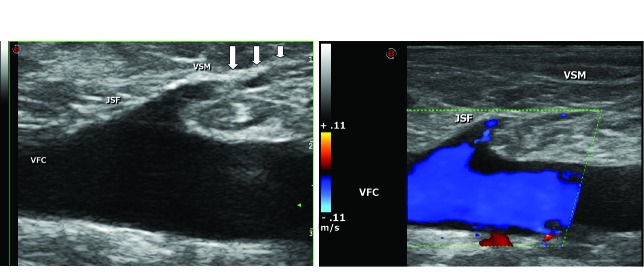
Aspecto fibrótico da veia safena magna na crossa, 1.360 dias após ablação com *endolaser* (seta). JSF = junção safeno-femoral; VFC = veia femoral comum; VSM = veia safena magna.

A taxa de sucesso global (oclusão permanente das veias tronculares) foi de 95,1% no período de seguimento. Em todos os casos com sucesso anatômico tardio pós-ablação, os achados ultrassonográficos registrados na última visita médica descrevem competência das junções safeno-femoral e safeno-poplítea, veias tronculares ocluídas com aspecto fibrótico e calibre muito reduzido e de difícil identificação (Figura 1).

Nos casos que evoluíram com recanalização (dois pacientes, gênero feminino, com insuficiência da VSM), os achados ultrassonográficos pós-operatórios, prévios à identificação da recanalização, descrevem insuficiência da junção safeno-femoral, VSM ocluída com conteúdo predominantemente hipoecogênico e retração venosa inferior a 50% do diâmetro inicial.

Os eventos adversos foram leves e autolimitados, com resolução espontânea entre 10 dias e 6 meses de pós-operatório (Tabela 6). Além disso, 39 pacientes (95,1%) apresentaram e mantinham uma melhora na classificação clínica CEAP na última revisão, em comparação à classificação clínica inicial.

**Tabela 6:** Eventos adversos.

**Eventos adversos**	**n (%)**
Pigmentação no trajeto venoso	4 (9,75%)
Fibrose/induração no trajeto venoso	2 (4,87%)
Parestesia transitória	3 (7,30%)
EHIT classe I*	1 (2,43%)
EHIT classe II*	1 (2,43%)
EHIT classe III*	0
EHIT classe IV*	0
TVP/TEP	0
Tromboflebite superficial	0
Infecção	0

EHIT: *endothermal heat-induced thrombosis*; TVP: trombose venosa profunda; TEP: tromboembolia pulmonar.

* Classificação descrita por Kabnick et al.^15^.

## DISCUSSÃO

Atualmente, a EVLA é considerada o padrão ouro para o tratamento da insuficiência venosa superficial troncular dos membros inferiores nos Estados Unidos e Reino Unido, sendo recomendada como terapia de primeira escolha^16^^-^ 18. Diversos trabalhos demonstram a alta eficiência da termoablação endovenosa, com elevadas taxas de sucesso técnico e baixos níveis de complicações, sobretudo com o *laser* com comprimento de onda (λ) de 1470 nm associado a fibra óptica de emissão radial^19^^-^ 22.

Além do consagrado *laser* de diodo com emissão de 1470 nm (meio ativo: InGaAsP), novos *lasers* com comprimentos de onda de interesse vêm surgindo também baseados em diodos semicondutores, como os diodos com comprimento de onda de emissão centrados em 1908 nm, 1920 nm e 1940 nm (meio ativo: AlGaIn)^8^^,^ 23. As vantagens práticas desses comprimentos de ondas são também advindas da elevada absorção da luz *laser* pela água intersticial presente na parede venosa, representada pelos respectivos coeficientes de absorção (μ_A_^água^) por esse cromóforo (Tabela 7)^24^. A situação de entrega da luz *laser* à parede venosa por fibra radial, com λ na região de 1940 nm, pode ser considerada uma evolução incremental ao atual estado da arte da técnica de termoablação endovenosa a *laser*, representado pelo *laser* 1470 nm com irradiação por fibra radial.

**Tabela 7:** Coeficiente de absorção da água na faixa NIR do espectro eletromagnético^24^.

**λ (nm)**	**Laser**	**µágua (cm-1)**	**Absorção** **1940 nm relativa**	**Absorção 1910 nm relativa**	**Absorção 1470 nm relativa**	**Absorção 980 nm relativa**
808	Diodo AlGaAs	0,02	5.991,50	4.517,00	1240,75	22,50
975	Diodo InGaAs	0,45	266,29	200,76	55,14	1
1064	Nd:YAG	0,12	998,58	752,83	206,79	3,75
1470	Diodo InGaAsP	24,815	**4,83**	**3,64**	1	0,018
1910	Diodo AlGaln	90,34	1,33	1	0,27	0,005
1940	Diodo AlGaln	119,83	1	0,75	0,21	0,004
2100	Ho:YAG	26,93	4,45	3,35	0,92	0,017

*λ*: comprimento de onda; µ_água_: coeficiente de absorção da água.

Efetivamente, vantagens importantes de comprimentos de onda situados na região *near-infrared* (NIR) do espectro óptico com relação à ablação endovenosa são o alto coeficiente de absorção pela água e a consequente baixa profundidade de penetração tecidual (confinamento da zona onde o calor é gerado no tecido exposto à radiação *laser*). Quanto maior o valor do coeficiente de absorção de um cromóforo (aqui, a água tecidual), função de λ, maior a quantidade de calor gerado em consequência de uma dada energia óptica aportada ao tecido e mais superficial (maior segurança); portanto, menor a quantidade de energia necessária para obtenção do dano térmico pretendido (termoablação)^25^.

Comparando-se os valores de μ_A_^água^ (λ) nesses novos comprimentos de onda com aquele de 1470 nm, temos μ_A_^água^ (1940 nm) = 4,83. μ_A_^água^ (1470 nm). Com relação a 1940 nm e 980 nm, o *laser* com λ = 1940 nm é aproximadamente 266 vezes mais absorvido pela água do que o *laser* λ = 980 nm e 4,8 vezes mais absorvido pela água do que o *laser* com λ = 1470 nm (Tabela 7)^24^. Devido ao maior coeficiente de absorção, LEEDs necessários com o *laser* 1940 nm são inferiores àqueles empregados para termoablação com *laser* 980 nm e 1470 nm. Além disso, os valores de penetração óptica efetiva na água, baseados em ambos os processos de absorção e de espalhamento, relativos a 1470 nm e 1940 nm, são aproximadamente 220 µ e 48 µ, respectivamente, em contraste com aquele em 980 nm (aproximadamente 3,0 mm)^24^^,^ 26 ^,^ 27.

Baseado nesses fatos, a ablação endovenosa com *laser* 1470 nm, procedida por irradiação tecidual homogênea (fibra radial) da parede venosa como alvo direto (cromóforo: água intersticial), representou, de fato, um grande avanço (*breakthrough*) na técnica de *endolaser*, com impacto de minimização dos efeitos adversos inerentes à técnica com outros *lasers* (808, 810, 940 e 980 nm, por exemplo)^9^^-^ 11 ^,^ 20 ^,^ 28. Por sua vez, a técnica com fibra radial e λ = 1940 nm representa um avanço incremental no estado da arte atual da termoablação a *laser*.

Nessa análise retrospectiva, as elevadas taxas de sucesso anatômico (obliteração) tanto imediato (100%) quanto tardio (95,1%) podem ser explicadas, do ponto de vista teórico, com as bases anteriormente expostas e que se aplicam, igualmente, ao *laser* com comprimento de onda em 1470 nm, produzindo resultados semelhantes^10^^,^ 11 ^,^ 20 ^,^ 29 ^,^ 30.

Um dado interessante que merece ser lembrado, relacionado ao *laser* com λ de 1940 nm em comparação ao *laser* com λ de 1470 nm, é o fato de ser quase cinco vezes mais absorvido pela água e com uma profundidade de penetração óptica efetiva de cerca de ¼ da profundidade de penetração do *laser* de 1470 nm^24^^,^ 26 ^,^ 27. Isso significa que quanto maior a absorção de fótons de um *laser* por um dado alvo contendo cromóforos absorvedores, maior é a quantidade de calor gerado e mais confinada é a zona de geração de calor, ou seja, a absorção dos fótons de um *laser* pelos cromóforos do tecido induz um aquecimento tecidual (aquecimento absorptivo). O calor absortivo (J/cm^3^), gerado *in situ,* é proporcional ao coeficiente de absorção μ_A_ (cm^-1^) multiplicado pela irradiância (W/cm^2^) e dependente linearmente do tempo de exposição^25^^,^ 31.

Enquanto o aporte térmico no tecido, oriundo da energia óptica absorvida e liberada na forma de calor, depende das propriedades ópticas do tecido e dos parâmetros da irradiação, como a irradiância e o tempo de exposição, o processo de difusão térmica condutiva é responsável pela transmissão (fluxo) de calor gerado pontualmente (fluxo de calor de uma região de maior temperatura para uma região de menor temperatura)^25^. Em outras palavras, o parâmetro governante de toda interação *laser*-tecido, relativo aos *lasers* de efeito fototérmico, é a temperatura. Quanto maior a absorção de fótons pelo cromóforo, maior é a quantidade de calor gerado e mais confinada é a geração do calor; porém, uma vez gerado, o calor se difunde do ponto de geração para as áreas termicamente mais frias, ou seja, a natureza e a extensão do dano térmico vão depender das propriedades ópticas do tecido (espalhamento e absorção), das propriedades térmicas do tecido (calor específico e condutividade térmica) e também, muito fortemente, dos parâmetros de exposição do *laser* (densidade de potência, tempo de exposição e densidade de energia).

O dano térmico ao colágeno tem um papel proeminente na ablação endovenosa nos resultados de curto e longo prazo. Biesman^32^ demonstrou que o colágeno se contrai com temperaturas próximas a 50˚C, mas a necrose de coagulação só ocorre com temperaturas entre 70 e 100˚C. Somente a administração de uma energia relativamente alta por unidade de comprimento resulta em temperaturas suficientemente elevadas para causar a desnaturação do colágeno^33^.

Na ablação endovenosa com *laser*, as temperaturas intraluminais podem elevar-se acima de 100ºC^28^^,^ 34 ^-^ 39, e estes perfis de temperatura são independentes do comprimento de onda, ou seja, o uso de diferentes comprimentos de ondas não influencia o perfil da temperatura endovenosa^28^^,^ 36. Por outro lado, considerando que as propriedades ópticas e térmicas dos tecidos, nesse caso, são as mesmas, a temperatura sofre forte influência dos parâmetros de exposição do *laser*.

Essas considerações nos fornecem, de certo modo, uma explicação racional para as duas falhas técnicas observadas neste estudo revisional. Em ambos os casos, a potência utilizada (Tabela 5) foi semelhante à potência média (4,1 W) empregada nos demais casos (Tabela 4). O diâmetro médio do segmento venoso tratado também não foi significativamente diferente da média dos diâmetros dos segmentos venosos tratados com sucesso em médio e longo prazo (Tabelas 3 e 5). O único parâmetro divergente dos casos bem-sucedidos foi a LEED, com uma média de 17 J/cm nos casos que recanalizaram e de 46,8 J/cm nos casos com sucesso anatômico, sendo essa diferença estatisticamente significativa (p = 0,0041).

Como já citado, a desnaturação do colágeno ocorre em temperaturas entre 70 e 100˚C; portanto, no processo de ablação é necessário administrar uma energia suficientemente alta para gerar temperaturas suficientemente elevadas para que o processo seja efetivo^33^. Nos dois casos em que houve recanalização, aproximadamente 12 meses após o tratamento endovenoso, a quantidade de energia entregue (LEED média = 17 J/cm) provavelmente não foi suficiente para aumentar a temperatura a ponto de produzir a desnaturação do colágeno. Esse aumento insuficiente da temperatura se traduziu nos achados ultrassonográficos já descritos e bem diferentes dos achados observados nos casos com sucesso anatômico.

Nenhum caso tratado com LEED superior a 30 J/cm (média: 46,8 J/cm) e diâmetro máximo de 10 mm, utilizando o *laser* com λ em 1940 nm, apresentou falha no tratamento durante o período de seguimento (média: 803 dias), com uma incidência muito baixa de eventos adversos, sem relevância clínica e autolimitados em sua totalidade.

Nesse ponto, torna-se absolutamente necessário compreender o conceito de extinção molar, que é a capacidade de uma substância absorver a luz a um dado comprimento de onda^33^. Devido ao coeficiente de extinção molar ser similar para a água e o sangue, quando se utiliza o *laser* com comprimento de onda de 1470 nm ou 1940 nm, é importante esvaziar a veia de sangue intraluminal^33^, pois, contrariamente, a maior parte da energia seria absorvida pelo sangue intraluminal, levando a uma oclusão trombótica e possível recanalização após poucos meses^27^^,^ 33 ^,^ 37 ^,^ 38.

Essa afirmativa baseia-se em estudo de Vuylsteke et al.^33^^,^ 40, em que foi avaliado o papel do sangue no resultado do tratamento endovenoso com *laser* de 1500 nm, verificando histologicamente o grau de destruição da parede venosa. Nesse estudo, concluíram que o volume de sangue intraluminal resulta em uma redução na destruição da parede venosa. A infiltração tumescente de líquido reduz a quantidade de sangue intraluminal, resultando em um aumento na destruição da parede venosa, além de atuar como um dissipador de calor prevenindo a destruição de tecidos perivenosos^40^. Segundo esses autores, a influência da tumescência sobre o diâmetro venoso é mais importante quando comparado com a posição de Trendelenburg.

Em síntese, o objetivo final do tratamento de varizes por termoablação a *laser* é a eliminação do refluxo patológico de sangue por oclusão durável ou permanente do lúmen venoso. De maneira geral, isso pode ser obtido pelo encolhimento da veia até que o lúmen venoso desapareça completamente ou por substancial dano no endotélio e na parede interna da veia, levando a uma oclusão secundária do lúmen por um coágulo, de maneira similar ao efeito produzido pelos agentes esclerosantes. A transferência substancial de calor para a parede da veia produz um significante encolhimento das fibras colágenas, com consequente redução do lúmen venoso. O montante de encolhimento parietal parece ser importante porque o lúmen remanescente, após o tratamento a *laser*, está sujeito a oclusão por formação de coágulo. Tardiamente, esse coágulo poderia estar sujeito a recanalização e poder-se-ia supor que quanto maior o diâmetro do coágulo, maior o risco de posterior recanalização^33^^,^ 40. Idealmente, após a termoablação a *laser*, a oclusão trombótica da veia safena é substituída por um cordão fibrótico que pode ser detectado frequentemente pelo ultrassom, mesmo anos após o procedimento (Figura 1).

## CONCLUSÃO

O *laser* 1940 nm mostrou-se muito seguro e efetivo, em médio e longo prazo, para os parâmetros utilizados, ou seja, potência média de 4,0 W e LEED acima de 30 J/cm (média de 46,8 J/cm) para a VSM e VSP com até 10 mm de diâmetro, independentemente da região tratada. Pode ser realizado em regime ambulatorial com anestesia local tumescente, com uma incidência muito baixa de eventos adversos, sem relevância clínica e autolimitados.
