# Supplementary material for: Resultados de médio e longo prazo do tratamento endovenoso de varizes com laser de diodo em 1940 nm: análise crítica e considerações técnicas
Source: J Vasc Bras. 2017 Jan-Mar;16(1):23–30. [Article in Portuguese] doi: 10.1590/1677-5449.010116 (PMC5829688; doi:10.1590/1677-5449.010116)
Supplement: Translation

# Translation: Medium and long-term outcomes of endovenous treatment of varicose veins with a 1940nm diode laser: critical analysis and technical considerations

**Authors:** Luiz Marcelo Aiello Viarengo, Gabriel Viarengo, Aline Meira Martins, Marília Wechellian Mancini, Luciana Almeida Lopes

**Affiliations:** 1 Clínica Viarengo – CV, Jundiaí, SP, Brazil.; 2 Núcleo de Pesquisa e Ensino de Fototerapia nas Ciências da Saúde – NUPEN, São Carlos, SP, Brazil.

**Keywords:** laser, varicose veins, endovenous ablation, thermoablation

## Abstract

**Background:**

Introduction of the endovenous laser technique for treatment of varicose veins triggered a efforts to identify an ideal wavelength, capable of producing the highest possible selective damage with the greatest safety and lowest incidence of adverse effects.

**Objectives:**

Assess medium to long term results of 1940nm diode laser treatment of varicose veins, correlating parameters used with durability of the anatomic outcome.

**Methods:**

This was a retrospective study of patients diagnosed with Chronic Venous Insufficiency at clinical stages CEAP C2 to C6 who underwent thermoablative treatment of trunk varicose veins using a 1940nm wavelength laser with a radial emission optical fiber, from April 2012 to July 2015. A systematic review was conducted of electronic medical records to obtain demographic and clinical data, including postoperative follow-up duplex ultrasound findings.

**Results:**

The average age of the 41-patient sample was 53.3 years and 37 patients were women (90.2%). The average follow-up time was 803 days. The average caliber of the treated veins was 7.8 mm. The immediate success rate was 100% with an average LEED of 45.3 J/cm. The late success rate was 95.1%, and two recanalizations were observed around 12 months after ablation. There was no recanalization in veins treated with a LEED greater than 30 J/cm.

**Conclusions:**

The 1940nm laser proved to be safe and effective in venous segments up to 10 mm in diameter, with the parameters proposed, over medium to long term time follow-up.

## INTRODUCTION

At many different centers worldwide, endovenous laser ablation (EVLA) has emerged as a standard for treatment of venous insufficiency and as a minimally invasive option for treatment of trunk varicose veins. Since its appearance, there have been continuous efforts to identify the ideal wavelength that will produce the maximum possible selective damage with the greatest margin of safety and the lowest incidence of adverse events. Many different wavelengths and different types of optical fibers have been tested in attempts to achieve this objective.^1^^-^ 8 Several authors have demonstrated that all of the wavelengths employed to treat varicose veins are equally capable of producing the desired anatomic results. The fundamental differences between different wavelengths are related to occurrence of adverse events.^9^^-^ 11

From the point of view of physics, the higher a tissue or a chromophore’s coefficient of light absorption, the greater the quantity of heat generated and the more confined the zone in which heat is generated. These principles indicate that a 1940nm laser would offer advantages, because it acts on water’s second largest absorption peak, theoretically increasing both the efficacy and safety of the procedure.

Durability is an important long-term characteristic of all vascular procedures. For varicose vein treatments, recurrence rates are potentially a valuable measure for assessing medium and long-term results of different venous disease treatment modalities.^12^^-^ 14

The objectives of this study are to conduct a retrospective analysis of the medium and long-term results of endovenous treatment of varicose veins using a 1940nm diode laser and to correlate the parameters used during treatment with durability of anatomic outcome (fibrotic occlusion or recanalization), clinical improvement according to the CEAP classification, and adverse events.

## METHODS

A retrospective review was conducted of patients diagnosed with chronic venous insufficiency at clinical stages C2 to C6 on the clinical, etiology, anatomical, and pathophysiology scale (CEAP), who had been treated endovenously for trunk varicose veins at a single center, using a laser with a wavelength of 1940nm (Medilaser, DMC, São Carlos, SP, Brazil), National Agency for Sanitary Vigilance [ANVISA] registration number 80030810129), with a radial emission optical fiber, from April 2012 to July 2015. The objective was to analyze medium and long-term results in terms of anatomic outcome (fibrotic occlusion, partial recanalization, and total recanalization), clinical improvement on the CEAP classification, and adverse events (pigmentation, thrombophlebitis, venous thrombosis, paresthesias, fibrous cord, and others). All data were anonymized.

The members of the study population were initially identified by searching electronic records for patients diagnosed with varicose veins, venous insufficiency, or venous hypertension (with or without ulceration) who had been treated with endovenous laser ablation of trunk varicose veins at least 12 months previously and had presented for the service’s routine clinical and ultrasonographic control examinations (at 7 days, 30 days, 3 months, 6 months, 9 months, 12 months, 18 months, 24 months, and annually thereafter). Patients with postthrombotic syndrome prior to surgical treatment or with reflux in the deep vein system were excluded.

Once approval had been granted by the Research Ethics Committee (ruling number 1.693.514), a systematic review was conducted of electronic medical records to obtain demographic data and clinical data, including duplex ultrasound findings during the postoperative follow-up period.

The demographic data analyzed included age, sex, race, and comorbidities. Clinical data included date of procedure, preoperative clinical diagnosis (CEAP classification), venous segment treated with laser ablation, extension and mean caliber of the venous segment treated, associated procedures, postoperative pain measured on an analog visual scale (AVS), intraoperative intercurrent conditions, immediate postoperative intercurrent conditions (within 30 days), late intercurrent conditions (with date of identification and progression), laser parameters employed (power, total energy and linear endovenous energy density [LEED], CEAP clinical classification at last clinical follow-up within study period, date of last follow-up for calculation of follow-up in days) and, additionally, information on the ultrasonographic findings from the last follow-up appointment attended within the study period, covering appearance of the ablated vein (fibrotic, fibroelastic, thrombotic, completely recanalized, partially recanalized, extension of recanalized segment, and competence or incompetence of saphenopopliteal and saphenofemoral junctions).

### Statistical analysis

Data were compiled in a table on Excel® 2011, version 14.3.6 (Microsoft, Redmond, WA, USA), and analyzed using Bioestat, version 5.3 (Instituto Mamirauá, Belém, PA, Brazil). Categorical variables were expressed as absolute and relative values and arranged in contingency tables. Quantitative variables were analyzed using descriptive statistics and expressed as means, standard deviations (SD), and maximum and minimum values. Intragroup differences were analyzed using Student’s *t* test. Results with p values < 0.05 were considered statistically significant.

## RESULTS

A total of 152 patients were identified who had been treated with thermoablation of trunk varicose veins between April of 2012 and July of 2015 using lasers of a number of different wavelengths. All procedures were conducted by the same surgeon, who has a great deal of experience with EVLA, in an ambulatory, extra-hospital setting with ultrasound-guided perivenous tumescent anesthesia combined with femoral nerve block, also guided with ultrasound. Of these, 50 limbs in 50 patients were treated with a 1940nm laser. Nine of these 50 cases that fit the study objective were excluded, two because follow-up records were available for less than 12 months, four because of postthrombotic syndrome prior to treatment, and three because of incomplete follow-up and data.

There were 37 (90.2%) female and 4 (9.8%) male patients and mean age was 53.3 years (range: 30 - 74 years; SD: 12.4 years). Mean number of gestations among the women was 2.2 (range 0 - 9; SD: 1.71). All demographic data are summarized in Table 1.

**Table 1:** Patients’ demographic data (n = 41).

**Mean age (range), years**	**53.3 (30-74)**
Sex	
Female	37 (90.2%)
Male	4 (9.8%)
Skin color	
White	34 (82.9%)
Black	7 (17.1%)
BMI (range)	26.1 (18.6-43.1)
Family history of venous disease	36 (87.8%)
Smoking	4 (9.8%)
Dyslipidemia	7 (17.1%)
Diabetes	3 (7.3%)
SAH	7 (17.1%)
Obesity	5 (12.2%)

BMI: body mass index; SAH: systemic arterial hypertension. Data are expressed as absolute values (%), except where indicated otherwise.

A total of 34 great saphenous veins (GSVs) (82.9%) and seven small saphenous veins (SSVs) (17.1%) were treated in 41 limbs of 41 patients, 19 right limbs (16 GSVs and 3 SSVs) and 22 left limbs (18 GSVs and 4 SSVs). Preoperative CEAP clinical classifications are summarized in Table 2. The anatomic parameters of the venous segments treated and the laser parameters employed are summarized in Tables 3 and 4 respectively.

**Table 2:** Preoperative clinical classification (CEAP).

	**C1**	**C2**	**C3**	**C4**	**C5**	**C6**
Overall (n = 41)	0	19	7	10	0	5
GSV* (n = 34)	0	18	6	8	0	2
SSV** (n = 7)	0	1	1	2	0	3

CEAP: clinical, etiology, anatomical, and pathophysiology; GSV: great saphenous vein; SSV: small saphenous vein.

* Patients with GSV incompetence only.

** Patients with SSV incompetence only.

**Table 3:** Anatomic parameters of veins ablated.

	**Mean Ø (mm)**	**Range (mm)**	**SD**	**Extension ablated (cm)**	**Range (cm)**	**SD**
GSV	7.82	(6.2 to 10.4)	1.08	48.1	(29 to 80)	11.9
SSV	7.14	(5.0 to 10.5)	2.03	24.3	(15 to 34)	6.2

GSV: great saphenous vein; SSV: small saphenous vein; SD: standard deviation.

**Table 4:** Laser treatment parameters.

	**Mean power (W)**	**Range (W)**	**SD**	**Mean LEED (J/cm)**	**Range (J/cm)**	**SD**
GSV	4.1	(3.5 to 7.0)	0.6	45.5	(15 to 104.7)	15.9
SSV	3.86	(3.0 to 4.0)	0.38	45.9	(35.8 to 54.6)	7.52
Overall	4.06	(3.0 to 7.0)	0.57	45.3	(15 to 104.7)	14.8

GSV: great saphenous vein; SSV: small saphenous vein; SD: standard deviation; LEED: linear endovenous energy density.

Primary ablation was achieved in 100% of cases. In all patients, phlebectomy of tributaries and varicose branches was conducted as a supplementary procedure. There were no intraoperative complications.

The mean length of postoperative follow-up was 803 days, varying from 467 to 1,360 days (SD: 291.3).

Intraoperative pain was rated on an AVS ranging from 0 to 10 (where 0 indicated “free from pain” and 10 “intense pain”). Twenty-three patients reported “free from pain” (56.1%), 17 patients (41.5%) chose “mild pain” (up to 3 points on the AVS), and one patient (2.4%) reported “moderate pain” (4 to 6 points on the AVS). In all cases, pain was related to phlebectomy.

Two recanalizations were observed during the follow-up period, one was a recanalization of the entire extension of a GSV (case 1), detected 421 days postoperatively; and the other was a recanalized infragenicular segment of a GSV (case 2), detected 342 days postoperatively (Table 5).

**Table 5:** Cases with late postoperative recanalization.

	**Time (days)**	**CEAP**	**Vein treated**	**Mean PO diameter** **(mm)**	**Power employed (W)**	**Mean LEED** **(J/cm)**	**BMI**
Case 1	421	C3	GSV	8.1	3.5	19.0	28.1
Case 2	342	C6	GSV	9.0	5.0	15.0	29.6

GSV: great saphenous vein; BMI: body mass index; PO: preoperative.

No recanalizations or treatment failures were observed in SSVs. Among the anatomically successful saphenous veins treatments (GSV + SSV), mean LEED was 46.8 J/cm (varying from 30.7 J/cm to 104.7 J/cm).

Overall success rate (permanent occlusion of trunk veins) was 95.1% over the follow-up period. In all of the cases of late post-ablation anatomic success, the ultrasonographic findings recorded at the last medical examination describe competence of the saphenofemoral and saphenopopliteal junctions, trunk veins occluded, with fibrotic appearance and a very reduced caliber, and difficult to identify (Figure 1).

**Figure 1:** Fibrotic appearance of the great saphenous vein at the arch, 1,360 days after endolaser ablation (arrow). CFV = common femoral vein; GSV = great saphenous vein; SFJ = saphenofemoral junction.

In the two cases that progressed to recanalization (two female patients with GSV incompetence), postoperative ultrasonographic findings prior to identification of the recanalization described saphenofemoral junction incompetence, GSV occluded with predominantly hypoechogenic content, and venous retraction of less than 50% of the initial diameter.

Adverse events were mild and self-limiting, with spontaneous resolution occurring from 10 days to 6 months after the procedure (Table 6). Additionally, 39 patients (95.1%) achieved and maintained to the last examination an improvement in CEAP clinical classification, compared with their initial clinical classifications.

**Table 6:** Adverse events.

**Adverse events**	**n (%)**
Pigmentation along vein trajectory	4 (9.75%)
Fibrosis/induration along vein trajectory	2 (4.87%)
Transitory paresthesia	3 (7.30%)
Class I EHIT *	1 (2.43%)
Class II EHIT *	1 (2.43%)
Class III EHIT *	0
Class IV EHIT *	0
DVT/PTE	0
Superficial thrombophlebitis	0
Infection	0

EHIT: endothermal heat-induced thrombosis; DVT: deep venous thrombosis; PTE: pulmonary thromboembolism.

* Classification described by Kabnick et al.^15^

## DISCUSSION

Currently, EVLA is considered the gold standard for treatment of insufficiency of superficial trunk veins in both the United States and the United Kingdom and is recommended as the first-choice treatment.^16^^-^ 18 Many different studies have demonstrated the elevated efficacy of endovenous thermoablation, with high rates of technical success and low levels of complications, particularly when a laser with a wavelength (λ) of 1470nm is used in combination with a radial emission optical fiber.^19^^-^ 22

In addition to the well-established diode laser with emission wavelength of 1470nm (active medium: InGaAsP), new semiconductor diode-based lasers with wavelengths of interest have been emerging, including emission wavelengths centered on 1908nm, 1920nm, and 1940nm (active medium: AlGaIn).^8^^,^ 23 The practical advantages of these wavelengths are also because of the high degree of absorption of laser light by the interstitial water in the vein walls, which can be illustrated by their respective absorption coefficients (μ_A_^water^) for this chromophore (Table 7).^24^ It can be considered that delivering laser light to the vein wall via a radial fiber at a λ in the region of 1940nm is an incremental development over the current state-of-the-art technique for laser endovenous thermoablation, which is the 1470nm laser with irradiation via a radial fiber.

**Table 7:** Coefficient of absorption in water in the NIR band of the electromagnetic spectrum.^24^

**λ (nm)**	**Laser**	**µwater (cm-1)**	**Relative absorption:** **1940nm**	**Relative absorption: 1910nm**	**Relative absorption: 1470nm**	**Relative absorption: 980nm**
808	AlGaAs diode	0.02	5,991.50	4,517.00	1240.75	22.50
975	InGaAs diode	0.45	266.29	200.76	55.14	1
1064	Nd:YAG	0.12	998.58	752.83	206.79	3.75
1470	InGaAsP diode	24.815	**4.83**	**3.64**	1	0.018
1910	AlGaln diode	90.34	1.33	1	0.27	0.005
1940	AlGaln diode	119.83	1	0.75	0.21	0.004
2100	Ho:YAG	26.93	4.45	3.35	0.92	0.017

*λ*: wavelength; µ_water_: coefficient of absorption in water.

The important advantages for endovenous ablation effectively offered by wavelengths within the near infrared (NIR) region of the optical spectrum are their high coefficients of absorption by water and the resultant shallow depth of penetration through tissues (which constrains the zone in which heat is generated in the tissues exposed to laser radiation). The higher the value of the absorption coefficient in a chromophore (in this case, water in tissues), which is a function of λ, the greater the quantity of heat generated for a given amount of optical energy supplied, the shallower the penetration (increasing safety), and, therefore, the lower the quantity of energy that is needed to produce the thermal damage intended (thermoablation).^25^

If we compare the μ_A_^water^ (λ) values offered by the new wavelengths with the figure for 1470nm, we find that μ_A_^water^ (1940nm) is 4.83 times the μ_A_^water^ (1470nm). Comparing the 1940nm and 980nm wavelengths, a laser at λ = 1940nm has an absorption in water approximately 266 times greater than a laser at λ = 980nm and 4.8 times greater than the absorption in water of a laser at λ = 1470nm (Table 7).^24^ The higher absorption coefficient means that the LEEDs needed when treating with a 1940nm laser are lower than those needed to achieve thermal ablation with a 980nm or even a 1470nm laser. Additionally, the values for effective optical penetration in water for 1470nm and 1940nm, based both on absorption and scattering processes, are approximately 220 µ and 48 µ respectively, in contrast with a value of approximately 3.0 mm for 980nm.^24^^,^ 26 ^,^ 27

Based on these facts, endovenous laser ablation at 1470nm, conducted by homogeneous tissue irradiation (with a radial fiber) with the vein wall as the direct target (chromophore: interstitial water) was indeed a major breakthrough for endolaser techniques, with impacts on minimization of the adverse effects inherent to the technique performed with other types of lasers (808, 810, 940 and 980nm, for example).^9^^-^ 11 ^,^ 20 ^,^ 28 In turn, conducting the technique with a radial fiber and λ = 1940nm is an incremental development of the state-of-the-art in laser thermal ablation.

In this retrospective analysis, the high rates of anatomic success (obliteration), both immediate (100%) and late (95.1%), can be explained from a theoretical perspective on the basis of the principles outlines above, which are applicable in the same way as with the 1470nm wavelength, producing similar results.^10^^,^ 11 ^,^ 20 ^,^ 29 ^,^ 30

One interesting feature that should be borne in mind in relation to the laser with a λ of 1940nm, compared with the laser at a λ of 1470nm, is that its light is absorbed almost five times more by water and it has an effective depth of optical penetration that is around one quarter of the penetration of the 1470nm laser.^24^^,^ 26 ^,^ 27 This is because the greater the absorption of the photons from a laser by a given target containing absorbent chromophores, the greater the quantity of heat generated and the more restricted the zone of heat generation, i.e., it is absorption of the photons from the laser by chromophores in the tissue that causes the tissues to heat up (absorptive heating). Absorptive heat (J/cm^3^), generated in situ, is proportional to the coefficient of absorption μ_A_ (cm^-1^) multiplied by the irradiance (W/cm^2^) and linearly dependent on exposure time.^25^^,^ 31

While the heat produced in the tissue, from the optical energy absorbed and released as heat, is dependent on the optical properties of the tissues and on the parameters of irradiation, such as irradiance and exposure time, the process of conductive thermal diffusion is responsible for transmission (flow) of heat generated locally (flow of heat from a higher-temperature region to a lower temperature region).^25^ In other words, the parameter that governs the entire interaction between laser and tissue, in relation to lasers that work by photothermal effects, is temperature. The greater the absorption of photons by the chromophore, the greater the quantity of heat generated and the more spatially restricted that generation of heat is; however, once generated, the heat diffuses from the site of generation to cooler areas. The result is that the nature and extent of thermal damage will depend on the optical properties of the tissue (scattering and absorption), the thermal properties of the tissue (specific heat and thermal conductivity) and also, very strongly, on the parameters of exposure to the laser (power density, exposure time, and energy density).

Thermal damage to collagen plays a preeminent role in endovenous ablation, affecting short and long-term results. Biesman^32^ demonstrated that collagen contracts at temperatures close to 50˚C, but that coagulation necrosis only occurs at temperatures from 70 to 100˚C. Only administration of a relatively high energy per unit of length results in sufficiently high temperatures to cause denaturing of collagen.^33^

During endovenous laser ablation, intraluminal temperatures can rise to over 100ºC,^28^^,^ 34 ^-^ 39 and these temperature profiles are independent of wavelength, i.e. use of different wavelengths does not influence the endovenous temperature profile.^28^^,^ 36 On the other hand, considering that the optical and thermal properties of the tissues, in this case, are the same, the temperature is strongly influenced by the laser exposure parameters.

To a certain extent, these considerations provide a rational explanation for the two technical failures observed in this review study. In both cases, the power employed (Table 5) was similar to the mean power (4.1 W) employed in the other cases (Table 4). Additionally, the mean diameter of the venous segment treated did not differ significantly from the mean diameters of the venous segments for which treatment was successful over the medium and long term (Tables 3 and 5). The only parameter that was different from the successful cases was LEED, at a mean of 17 J/cm in the recanalization cases, compared to 46.8 J/cm in cases in which anatomic success was achieved, which is a statistically significant difference (p = 0.0041).

As already mentioned, denaturing of collagen occurs at temperatures from 70 to 100˚C; therefore, it is necessary to administer sufficient energy during the ablation process to generate high enough temperatures to make the process effective.^33^ In both the cases in which there was recanalization (at approximately 12 months after endovenous treatment), it is probable that the quantity of energy delivered (mean LEED = 17 J/cm) was not enough to increase the temperature to the point at which denaturing of collagen would occur. This insufficient increase in temperature was translated into the ultrasonographic findings described, which were very different from the findings observed in cases with anatomic success.

None of the case treated with a 1940nm λ laser using a LEED greater than 30 J/cm (mean: 46.8 J/cm) for a maximum diameter of 10 mm resulted in treatment failure detected during the follow-up period (mean: 803 days). The incidence of adverse events was very low, and all of those that did occur were not clinically relevant and were self-limiting.

At this point, it is absolutely necessary to understand the concept of molar extinction, which is a substance’s capacity to absorb light of a given wavelength.^33^ Since the coefficient of molar extinction is similar for water and blood, when a laser with a wavelength of 1470nm or 1940nm is used, it is important to empty the vein of intraluminal blood,^33^ because otherwise the majority of the energy will be absorbed by the intraluminal blood, leading to thrombotic occlusion and possible recanalization after a few months.^27^^,^ 33 ^,^ 37 ^,^ 38

This statement is based on a study by Vuylsteke et al.,^33^^,^ 40 that assessed the role played by blood in the results of endovenous treatment with a 1500nm laser, histologically evaluating the degree of destruction of the vein wall. The study concluded that the volume of intraluminal blood results in a reduction of vein wall destruction. Tumescent infiltration of liquid reduces the quantity of intraluminal blood, resulting in an increase in vein wall destruction, in addition to acting to dissipate the heat, preventing destruction of perivenous tissues.^40^ According to the authors, the influence of tumescence on venous diameter is more important than the Trendelenburg position.

In summary, the final objective of treating varicose veins with laser ablation is to eliminate pathological reflux of blood by durable or permanent occlusion of the vein lumen. In general, this can be achieved by shrinking the vein until the lumen disappears completely or by substantial damage to the endothelium and the internal wall of the vein, leading to secondary occlusion of the lumen by a clot, in a similar manner to the effect produced by sclerosing agents. Substantial transfer of heat to the vein wall reduces on significant shrinkage of its collagen fibers, with a consequent reduction in the lumen. The magnitude of parietal shrinkage appears to be important because the lumen that remains after laser treatment is subject to occlusion by clot formation. Later, this clot may be subject to recanalization and it can be supposed that the greater the diameter of the clot, the greater the risk of recanalization later.^33^^,^ 40 Ideally, after laser thermal ablation, thrombotic occlusion of the saphenous vein is substituted by a fibrous cord that can often by detected by ultrasound, even years after the procedure (Figure 1).

## CONCLUSIONS

The 1940nm laser proved to be very safe and effective over the medium and long term with the parameters employed, i.e. a mean power of 4.0 W and a LEED greater than 30 J/cm (mean of 46.8 J/cm) for GSVs and SSVs with diameters of up to 10 mm, irrespective of the region treated. It can be conducted in an ambulatory setting using tumescent local anesthesia, with a very low incidence of adverse events, which are without clinical relevance and are self-limiting.
